# Case report of immune checkpoint inhibitor induced cholestatic hepatitis, acute renal injury and asymptomatic pancreatic enzyme elevation simultaneously

**DOI:** 10.3389/fimmu.2025.1679328

**Published:** 2025-11-19

**Authors:** Rui Jiao, Chenyu Wang, Hongyan Ying, Muwen Nie, Jing Leng, Yuan Liu, Zhiyang Zhang, Na Zhou, Chunmei Bai

**Affiliations:** 1 Peking Union Medical College Hospital, Department of Oncology, Beijing, China; 2 Peking Union Medical College Hospital, Department of Internal Medicine, Beijing, China

**Keywords:** cholestatic hepatitis, steroid-refractory toxicity, artificial liver therapy, immune checkpoint inhibitors, multi-organ immune-related adverse events

## Abstract

**Background:**

Immune checkpoint inhibitors (ICIs) have demonstrated promising antitumor activity. However, it may induce immune-related adverse events (irAEs). Multi-organ irAEs remain heterogeneous and incompletely characterized. We report a unique irAE pattern with synchronous hepatic, renal and pancreatic involvement which is first reported.

**Case presentation:**

5 males developed a rare multi-organ irAE pattern with concurrent cholestatic hepatitis, renal injury and pancreatic enzyme elevation, representing 0.40% of the ICI-treated cohort. The syndrome showed early, rapidly onset and often began with nonspecific complaints requiring close monitoring.

**Management:**

Responses of high dose glucocorticoid were varied by organs. Compared with renal and pancreatic injury, cholestatic hepatitis was less responsive to steroids. Out of 3 cases who were refractory to steroids, 2 cases were given artificial liver treatment and 1 case was given bilirubin adsorption after steroid failure.

**Outcomes:**

Immunosuppression by standard glucocorticoid showed limited efficacy in cholestatic hepatitis. The 2 patients received artificial liver support improved biochemically, while bilirubin adsorption alone provided only transient reduction of bilirubin in one case. As for final clinical outcome, one patient died from severe infection during therapy agianst irAE; one patient died from cancer progression despite irAE recovery; and the others achieved full recovery from this irAE combination.

## Introduction

With widespread application of immune checkpoint inhibitors (ICIs), irAEs have emerged as a critical safety concern. About 5%-9% of patients receiving anti-PD-(L)1 therapy experienced multisystem irAEs (1–3). However, multisystem irAEs exhibit heterogeneity, depending on tumor type and organs involved. Pneumonitis/thyroiditis, hepatitis/thyroiditis, dermatitis/pneumonitis and dermatitis/thyroiditis were common multi-organ irAE patterns (1). However, no cases of simultaneous hepatic, renal, and pancreatic injury associated with ICIs have been documented. To our knowledge, this study is the first to report this unique combination of irAEs. We aim to enhance clinicians’ awareness of this irAEs syndrome and summarize our treatment insights.

## Case presentations

### Case 1

**Figure 1:**
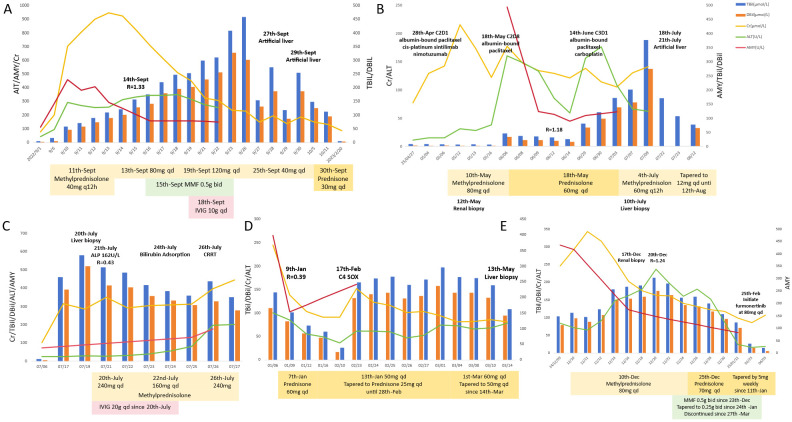
Clinical course of **(A)** Case 1, **(B)** Case 2, **(C)** Case 3, **(D)** Case 4 and **(E)** Case 5.

A 50-year-old male was diagnosed with esophageal cancer (AJCC 8th edition cT3N3M0, stage IVA) in July 2022. He had past medical history of asymptomatic kidney stones. He underwent two cycles of docetaxel and cisplatin combined with tislelizumab (200mg intravenously every 3 weeks).The 2^nd^ cycle treatment was initiated on Sept 1^st^, 2022. 4 days following the administration of the 2^nd^ cycle (September 5^th^), the patient exhibited fatigue, nausea, and vomiting and decreasing urinary output volume (500 ml/day). Biochemical analysis on Sept 14^th^ revealed: creatinine (Cr) 703 μmol/L (baseline 78 μmol/L), alanine aminotransferase (ALT) 292 U/L, total bilirubin(TBil) 58.9 μmol/L, direct bilirubin (DBil) 47.4 μmol/L, amylase (AMY) 459 U/L (ULN 125 U/L) and lipase (LIP) 150 U/L (ULN 78 U/L). The ratio of ALT to ALP [R = (ALT/ULN)/(ALP/ULN)]was 1.33. Excluding of infectious etiologies, biliary obstruction, and renovascular abnormalities (**Table 1**), the diagnosis of immune-related hepatitis [Common Terminology Criteria for Adverse Events (CTCAE) grade 3 (G3)], nephritis(G3), and pancreatic enzyme elevation (G2) was confirmed. Intravenous methylprednisolone was initiated at 40 mg twice daily (equivalent to prednisone 1.64 mg/kg/day for 61kg body weight). After one week of corticosteroid, creatinine, amylase, transaminase began to decrease and the patient’s urine output increased. However, bilirubin levels progressively increased with more apparent jaundice. Despite treatment escalation with mycophenolate mofetil (0.5g twice daily for a week), intravenous immunoglobulin (10g daily for 5 days), and high-dose methylprednisolone (120 mg daily), the hyperbilirubinemia proved refractory, showing progressive elevation. Artificial liver with models of plasma perfusion (PP), plasma exchange (PE), dual-plasma molecular adsorption system (DPMAS) and plasma diafiltration (PDF) was performed twice on Sept 27^th^ and 29^th^, respectively, which resulted in a marked and sustained reduction in serum bilirubin and resolution of jaundice. Steroid was tapered from 30 mg daily after artificial liver and reduced by 5 to 10 mg a week. Following the voluntary discontinuation of antitumor therapy, the patient experienced a tumor recurrence in February 2023, which resulted in a tracheoesophageal fistula. The patient died on May 28^th^, 2023. The timeline and comprehensive treatment are provided in **Figure 1A**, **Supplementary Figure 1A**.

**Table 1:** Pathology, imaging examinations and screening for infection and immunity abnormalities.

Case Number	Case 1	Case 2	Case 3	Case 4	Case 5
Pathology					
Mismatch repair	None	None	pMMR ^a^	None	pMMR
PD-L1 (22C3) CPS^b^	None	3	5	None	15
Imaging					
Ultrasound and computed tomography (CT)	No hydronephrosis and ureteral dilation No extrahepatic biliary duct dilation No pancreatic enlargement with ill-defined margins or heterogeneous parenchymal density				
Infection					
Anti-HAV IgM	Neg	Neg	Neg	None	None
HBsAg	Neg	Neg	Neg	Neg	Neg
HBsAb	Neg	Neg	Neg	Neg	Neg
HBeAg	Neg	Neg	Neg	Neg	Neg
HBeAb	Neg	Neg	Neg	Positive	Neg
HBcAb	Neg	Neg	Neg	Positive	Neg
HCV-Ab	Neg	Neg	Neg	Neg	Neg
Anti-HEV IgM	Neg	Neg	Neg	Neg	None
CMV -DNA (copies)	Neg	844	Neg	Neg	Neg
EBV -DNA (copies)	Neg	Neg	Neg	Neg	Neg
SARS-CoV-2 RNA	Neg	Neg	Neg	Neg	Neg
Immunology					
Autoimmune hepatitis antibodies^c^	Neg	Neg	Neg	Neg	ANA S1:80^d^
ANCA^e^	Neg	Neg	Neg	Neg	Neg
Anti-GBM-Ab^f^	Neg	Neg	Neg	Neg	None

a : Proficient mismatch repair.

b : PD-L1 IHC 22C3 pharmDx combined positive score.

c : Including ANA (antinuclear antibody), ACA (anticardiolipin antibody), SMA (anti-smooth muscle antibody), AMA (anti-mitochondrial antibodies), GP210 (anti-glycoprotein-210 antibody), SP100 (anti-soluble acidic nuclear protein of 100kDa antibody), SLA (soluble liver antigen), and LKM-1 (liver-kidney microsomal 1 antibodies), and LC-1 (liver cytosol type 1 antibodies).

d : Anti-neutrophilcytoplasmic antibody speckled type 1:80 positive.

e : Anti-neutrophilcytoplasmic antibody, which including perinuclear ANCA-IgG, cytoplasmic ANCA-IgG, PR3 (proteinase 3)-ANCA, MPO (myeloperoxidase)-ANCA.

f : Anti-Glomerular Basement Membrane Antibody.

### Case 2

A 65-year-old male was diagnosed with esophageal cancer (cT3N2M0, stage III) in March 2025. He began to receive TP regimen combined with sintilimab and nimotuzumab on Apr 4 (albumin-bound paclitaxel 200mg on day 1,8, cisplatin 120mg on day 1, sintilimab 200mg on day 1, nimotuzumab 400mg on day 1,8,15, every 3 week). The 2^nd^ cycle treatment was started on Apr 28^th^. The patient subsequently developed fatigue and poor appetite, and serum creatine increased to 324umol/L (May 10^th^) in two weeks, with normal bilirubin. Renal biopsy demonstrated interstitial nephritis with lymphocytic infiltration. Intravenous methylprednisolone 80 mg daily (1.67 mg/kg/day prednisone-equivalent) was initiated on May 10^th^. Cr level decreased to 130 μmol/L after one week steroid treatment and the patient reported improvement in symptoms of nausea and anorexia. The patient continued to receive albumin-bound paclitaxel 200mg and the steroid was tapered to oral prednisone 60 mg daily on May 18^th^. Two weeks later, the patient developed choluria after prednisone tapering to 45mg daily (Cr 177 μmol/L, TBil 45.5 μmol/L, DBil 33.4μmol/L, ALT 160U/L, AMY 493U/L, LIP 61U/L). After intensification of prednisone to 60mg daily, which led to a reduction in creatine to 121μmol/L and TBil/DBil to 25/16umol/L, chemotherapy was reinitiated on June 14^th^. However, hepatic dysfunction recurred following chemotherapy with TBil/DBil rising from 121/98umol/L (Jun, 30^th^) to 376/274umol/L (Jul, 9^th^), (R value 0.48). The liver biopsy showed: (a) cholestasis, (b) scattered lymphocytes in hepatic cords, and (c) focal interface hepatitis with lymphocytic infiltration in portal areas The diagnosis comprised immune-related hepatitis (G3), nephritis (G2), and pancreatic enzyme elevation (G1). Intensified steroid dosing failed to attenuate bilirubin elevation. The patient received artificial liver on July 18^th^ and July 22^th^. The total bilirubin decreased to 170μmol/L successfully on July 22^th^ and the patient reported improvement in choluria. No obvious adverse effects observed after the treatment of steroid and artificial liver. The timeline and comprehensive treatment are provided in **Figure 1B**, **Supplementary Figure 1B**.

### Case 3

**Figure 2:**
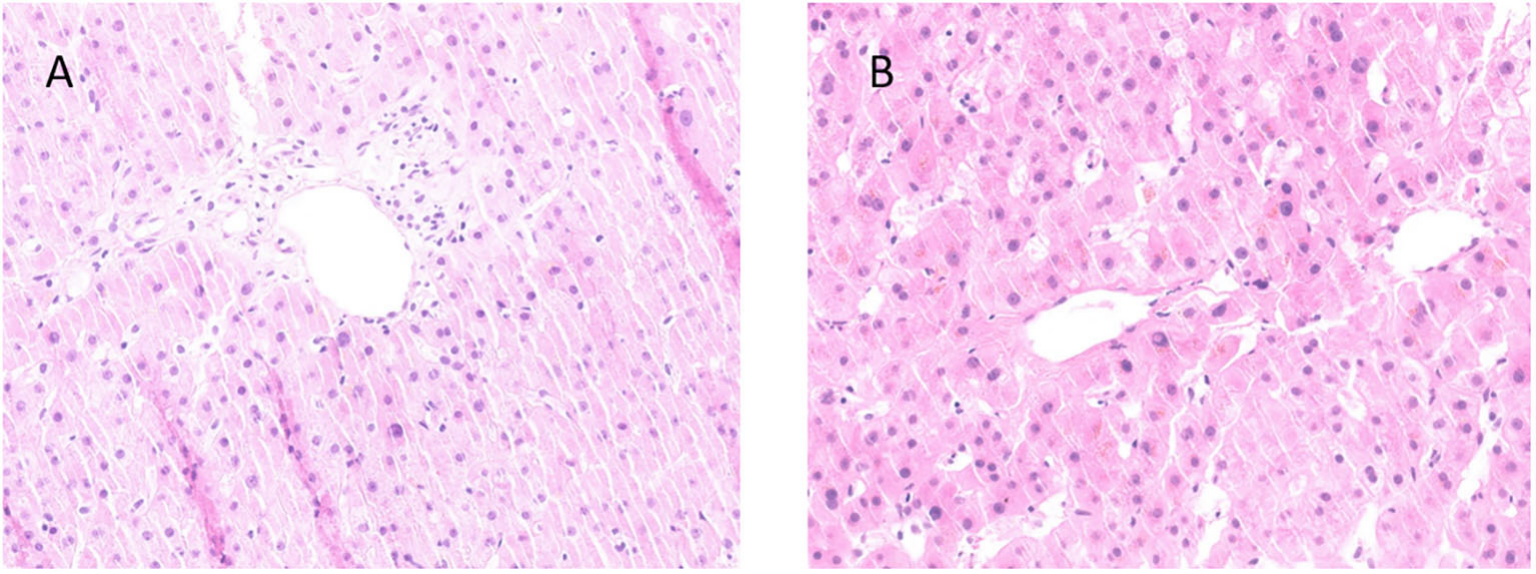
Liver biopsy specimens from Case 3 and Case 4. **(A)** Case 3: Hepatocyte degeneration with intrahepatic cholestasis, multifocal necrosis and portal lymphocytic infiltration with mild fibrosis. **(B)** Case 4: diffuse hepatocyte ballooning, cholestasis, focal necrosis with preserved lobular architecture, and mild portal fibrosis with bile ductular reaction (the biopsy was performed after one month of steroid).

The case involved a 70-year-old male who presented in May 2023 with histologically confirmed stage IIIA (cT4aN2M0) gastric adenocarcinoma. The patient had no significant comorbidities. He received two cycles of tislelizumab combined with SOX regimen (oxaliplatin 200 mg on day 1, tegafur 60 mg twice daily over day 1 to day 14, tislelizumab 200 mg on day 1, every 3 weeks). After the first cycle of treatment, the patient developed cutaneous irAE manifesting as erythematous papules with blistering on extremities (G2), which resolved following topical halometasone and oral cetirizine. Tislelizumab was discontinued after 2^nd^ cycle (June 9^th^, 2023) due to progressive skin toxicity, while 3^rd^ cycle SOX regimen continued. On July 10^th^, 2023, the patient developed nausea, anorexia, scleral jaundice and oliguria (urine volume was 800 ml/day). Laboratory results showed: Cr 314 μmol/L (baseline 102 μmol/L), ALT 25 U/L, TBil 459.7 μmol/L, DBil 391.7 μmol/L, AMY 131 U/L, LIP 237 U/L, and the R value was 0.43 on July 21^st^. Liver biopsy (**Figure 2A**) was performed, which demonstrated hepatocyte degeneration with intrahepatic cholestasis, multifocal necrosis and portal lymphocytic infiltration (predominantly T-cells) with mild fibrosis. Excluding other potential causes (**Table 1**), immune-related hepatitis (G3), acute kidney injury (G3) and asymptomatic elevated pancreatic enzymes (G1) was diagnosed and methylprednisolone 240 mg daily (3.75 mg/kg/day prednisone-equivalent for 80 kg) was initiated. Despite subsequent IVIG administration (20g daily for 5 days since July 20^th^) and bilirubin adsorption (July 24^th^), his liver function and oliguria (300ml/d) worsened and continuous renal replacement therapy was applied on July 26^th^. Unfortunately, these irAEs failed to improve and was complicated with severe infection and progressive dyspnea. The patient felt dyspnea and developed coma on July 27^th^, 2023. Then bronchoscopy demonstrated invasive pulmonary aspergillosis. The patient succumbed to septic shock on the same day. The timeline and comprehensive treatment are provided in **Figure 1C**, **Supplementary Figure 1C**.

### Case 4

A 51-year-old male who was diagnosed with stage IV gastric cancer (cT4bN3bM1) in September 2022. He received 3 cycles of Sintilimab plus SOX regimen from October 28^th^ to December 28^th^, 2022. He also had a medical history of past HBV infection (HBsAg^-^/HBsAb^+^/HBcAb^+^). He developed fatigue, anorexia, and choluria afterwards. Laboratory tests on January 6^th^, 2023 revealed elevated ALT (100 U/L), TBil (143.8 μmol/L), DBil (110.6 μmol/L), ALP (631 U/L), Cr (245 μmol/L, baseline 66μmol/L), AMY (398 U/L), LIP (348 U/L). R value was 0.39 on Jan 6^th^. The patient was diagnosed with ICIs-induced hepatitis (G3), acute kidney injury (G2), and asymptomatic pancreatic enzyme elevation (G2). Prednisolone 60mg daily (1mg/kg/day) was initiated on the following day and the levels of bilirubin, ALT and creatinine began to decline. The patient reported improvement in symptoms of fatigue, anorexia, and choluria. The steroid was tapered to 50mg daily on January 13^th^, followed by weekly reduction of 5mg. By Feb 10^th^, bilirubin, ALT and creatinine levels returned to normal. Sintilimab was discontinued, while the 4^th^ cycle of chemotherapy with SOX regimen was administered on Feb 17^th^. However hepatic and renal dysfunction recurred. The prednisolone was intensified to 60 mg daily since Mar 1^st^. Liver biopsy (**Figure 2B**) performed on Mar 13^th^ revealed diffuse hepatocyte ballooning, cholestasis, focal necrosis with preserved lobular architecture, and mild portal fibrosis with bile ductular reaction, without significant lymphocyte infiltration in the biopsy specimens. The prednisolone was tapered to 50mg daily on Mar 14^th^. However, the tumor progressed rapidly. He died on Apr 13^th^, 2023. The timeline and comprehensive treatment are provided in **Figure 1D**; **Supplementary Figure 1D**.

### Case 5

A 65-year-old male with a medical history of well-controlled type 2 diabetes diagnosed with stage IVA lung cancer (cT4N2M1a) in October 2024. He received pemetrexed and cisplatin regimen on October 24^th^. Next-generation sequencing (NGS) identified an epidermal growth factor receptor (EGFR) exon 20 insertion (EX20ins). Tislelizumab 200 mg was added in the 2^nd^ cycle treatment on Nov 23^rd^, 2024. Two weeks after the first dose of tislelizumab, he developed anorexia and oliguria (urine volume 500 ml/day). Laboratory investigations demonstrated elevated levels of ALT (84 U/L), TBil (103.5 μmol/L), DBil (79.5 μmol/L), ALP (448 U/L), Cr (246 μmol/L; baseline 78 μmol/L), AMY (435 U/L), and LIP (85 U/L). R value was 1.24. Renal biopsy performed on demonstrated interstitial nephritis with dense lymphocytic infiltration. The diagnosis of ICIs-related hepatitis (G3), nephritis (G3), and asymptomatic pancreatic enzyme elevation (G2) was established. Intravenous methylprednisolone 80 mg daily (1.6 mg/kg/day prednisone-equivalent) was initiated on Dec 16^th^, 2024. Due to unsatisfied response of bilirubin to corticosteroids, MMF 0.5g twice daily was added since Dec 23^rd^ and the level of bilirubin began to significantly decline since Dec 26^th^. The steroid was tapered to predsolone 70 mg daily on Dec 25^th^, followed by weekly reductions of 5 mg since January 11^th^, 2025. The MMF was tapered to 0.25g twice daily on Jan 24^th^ and discontinued since Mar 27^th^. The patient reported improvement in anorexia and oliguria. After recovery, he restarted treatment with furmonertinib (a EGFR tyrosine kinase inhibitor) 80 mg daily (Feb 25^th^, 2025), escalated to 120 mg daily on Mar 1^st^. No significant adverse effects were observed after the treatment of steroid and MMF. Serial imaging assessments (Apr and June, 2025) demonstrated stable disease. The timeline and comprehensive treatment are provided in **Figure 1E**; **Supplementary Figure 1E**.

From Mar 1^st^, 2020 to Mar 31^th^, 2025, 1239 patients had received at least one dose ICIs in Department of Medical Oncology at Peking Union Medical College Hospital, A total of 5 patients (all males) were diagnosed with this kind of multiorgan irAE syndrome, accounting for 0.40% of the ICI-treated population.

## Discussion

With the expanding application of immune checkpoint inhibitors (ICIs), irAEs have become a major concern, demonstrating unpredictable onset patterns and effected organs (4). The incidence of irAEs reported in different studies ranges from 15.34% to 85.23%, most frequently affected sites were cutaneous (27.0%-56.1%), endocrine (0.8%-30.4%), and gastrointestinal (0%-33.8%) system (5, 6). Incidence of ICI-induced hepatitis was reported to be 0.7% to 2.1% in PD-1, and much higher in CTLA-4 population (up to 12-16%), notably, fulminant hepatic failure was relatively rare (0.1%‐0.2%) (7). Acute renal injury (AKI), and pancreatis occured with an incidence of approximately 2-3% and 0.3-3.9% (8–11), respectively. The severe ICI-related AKI (defined as serum creatinine elevation > 3×baseline, or SCr >4.0 mg/dL, or requiring renal replacement therapy) was 0.6% (12). Multi-organ irAEs are relatively uncommon (5-9%), pneumonitis and thyroiditis emerged as the most frequently observed combination (13). This case series represents the first documented description of a distinct multi-organ irAE syndrome characterized by synchronous involvement of hepatic, renal, and pancreatic system, with an incidence rate of 0.40% (5/1239) in the Department of Medical Oncology at Peking Union Medical College Hospital. The mechanism of multisystem irAEs was still unknown, it may be attributed to shared pathobiological features, such as specific human leukocyte antigen (HLA) profiles or autoantibody generation (14, 15).

Checkpoint inhibitor-induced liver injury (CHILI) typically develops within 1–3 months, ICI-AKI (ranging from 1 to 10 months) and ICI-pancreatitis (from 1 to 13 months) demonstrate variable latency (7, 16, 17). In this study, the median time from initiation and last dose of ICI to symptom onset/lab abnormalities was 30 days (range 16-65) and 6 days (range 4-28), indicating early onset and rapid progression of this unique syndrome. Besides, the onset was insidious, with initial nonspecific symptoms (nausea, fatigue, anorexia or oliguria) requiring high clinical vigilance and necessitating close monitoring.

In our case series, compared with renal and pancreatic injury, liver injury is more refractory to treatment. Approximately 40% of patients with immune-related AKI could achieve complete recovery following corticosteroid therapy (18). Asymptomatic pancreatic enzyme elevation (G1, G2) did not need corticosteroid in our case series. CHILI can be classified into three types: cholestatic, hepatocellular, and mixed type (19, 20). Liver biopsy is not a mandatory requirement for the routine diagnosis of CHILI, which could be typed by R [(ALT/ULN)/(ALP/ULN)] (21). Cholestatic type CHILI was characterized by high ALP levels (R < 2) and jaundice, with CD8+ T cell infiltration in the biliary tract as pathological manifestation (20). All 5 patients in this study showed R < 2 (range from 0.39 to 1.33, median 0.48), indicating biliary stasis-type CHILI. Some studies indicated that 37.5–50% of acute CHILI patients can improve without corticosteroid therapy (22, 23). However, compared to hepatocellular type, cholestatic type patients had a poorer response with 11.5% in previous study, and increasing corticosteroid dosage did not significantly improve liver damage (24). For steroid-refractory patients, majority of them (82.9%) had received MMF, other therapeutic attempts included infliximab, gamma globulin, rituximab (25). In our study, only patient No.4 with moderate elevation of bilirubin achieved complete recovery of liver function after glucocorticoid and patient No.5 showed efficacy after adding MMF when hepatic irAE resistant to glucocorticoid. The above treatment demonstrated limited efficacy in fulminant hepatitis in the other 3 patients (No.1, 2, 3). In this study, 2 patients (patient No.1 and No.2) showed liver function improvement after artificial liver therapy, bilirubin adsorption provided only transient reduction in serum level without liver function recovery (patient No.3). Our artificial liver therapy mainly included plasma exchange (PE) and double plasma molecular adsorption system (DPMAS). In some cases, ICI related fulminant hepatitis was successfully treated with PE, a preferred treatment for some immune-mediated diseases, which can accelerate the removal of ICIs and have the ability to increase Treg cells (26–28). DPMAS can reduce serum bilirubin levels, clear inflammatory factors, reduce inflammatory response syndrome, block the progression of liver failure, and improve the prognosis of liver failure (29, 30).

Relationship between severe irAEs and survival remains controversial, high-dose corticosteroids may increase infection risk and suppress antitumor immunity (31, 32). Patient No.3 died from severe infection, while patient No.1 who received prolonged high-dose steroid, died due to rapid disease progression though recovery from irAEs. Based on these experience, artificial liver therapy may be considered to be used as early intervention in severe CHILI patients who develop progressively or glucocorticoid resistant, reducing corticosteroid requirements and mitigating the risk of immunosuppression.

Tofacitinib, a janus kinase - signal transducer and activator of transcription (JAK-STAT) inhibitor, has showed efficacy in treating autoimmune diseases. Clinical studies have reported remarkable remission rates, achieving 96.7% in steroid-resistant patients and 100% in patients with steroid taper failure (33). Previous case reports have demonstrated the efficacy of JAK inhibitors in the treatment of hepatocellular hepatitis (34). Though, the therapeutic efficacy of tofacitinib for patients with cholestatic CHILI remains undetermined and needs further investigation.

Though ICI discontinued, patient No.4 experienced recurrent liver and kidney dysfunction after chemotherapy following irAE recovery, while one lung cancer patient successfully attempted EGFR targeted therapy after irAE recovery and achieved stable disease without dysfunction of liver and kidney. It is significant to choose appropriate timing for treatment resumption after irAE recovery, maybe it needs extended recovery intervals for patients with this type of irAEs.

This study has several limitations. It is single-center design and exclusively male cohort may limit the generalizability of the findings. Furthermore, the case series was predominantly composed of gastrointestinal tumors (with only one lung cancer case), which may further restrict the applicability of our conclusions to other cancer types.

## Patient perspective

Patients described abrupt onsets with non-specific symptoms prompting urgent hospital evaluation. Those receiving artificial liver support reported rapid jaundice relief as a turning point. Families emphasized anxiety around infection during high-dose steroids. Patients favored clear timelines for steroid taper and close outpatient monitoring after discharge.

## Conclusion

In summary, this case series showed a rare condition of multisystem irAEs involving hepatitis, acute renal injury, and asymptomatic pancreatic enzyme elevation. Compared to renal and pancreatic injury, the therapeutic challenge was steroid-refractory cholestatic liver injury. The majority of these patients experienced poor clinical outcomes attributable to infections, recurrent hepatic/renal dysfunction, or rapid tumor progression. Early glucocorticoid initiation and early intervention of artificial liver treatment are significant. From our perspective, ICIs should be discountinued, and optimal timing of other anti-cancer therapy (such as chemotherapy) need to be explored in further study.

## Data Availability

The original contributions presented in the study are included in the article/**Supplementary Material**. Further inquiries can be directed to the corresponding authors.

## Supplementary material

Supplementary Figure 1 Timeline for disease onset, treatment interventions, and outcomes of **(A)** Case 1, **(B)** Case 2, **(C)** Case 3, **(D)** Case 4 and **(E)** Case 5.

## References

[1] Shankar B Zhang J Naqash AR Forde PM Feliciano JL Marrone KA . Multisystem immune-related adverse events associated with immune checkpoint inhibitors for treatment of non-small cell lung cancer. JAMA Oncol. (2020) 6:1952–6. doi: 10.1001/jamaoncol.2020.5012, PMID: 33119034 PMC7596677

[2] Kichenadasse G Miners JO Mangoni AA Rowland A Hopkins AM Sorich MJ . Multiorgan immune-related adverse events during treatment with atezolizumab. J Natl Compr Cancer Netw: JNCCN. (2020) 18:1191–9. doi: 10.6004/jnccn.2020.7567, PMID: 32886899

[3] Laparra A Kfoury M Champiat S Danlos FX Martin-Romano P Simonaggio A . Multiple immune-related toxicities in cancer patients treated with anti-programmed cell death protein 1 immunotherapies: a new surrogate marker for clinical trials? Ann Oncol. (2021) 32:936–7. doi: 10.1016/j.annonc.2021.04.006, PMID: 33865965

[4] Puzanov I Diab A Abdallah K Bingham CO 3rd Brogdon C Dadu R . Managing toxicities associated with immune checkpoint inhibitors: consensus recommendations from the Society for Immunotherapy of Cancer (SITC) Toxicity Management Working Group. J Immunother Cancer. (2017) 5:95. doi: 10.1186/s40425-017-0300-z, PMID: 29162153 PMC5697162

[5] Socinski MA Jotte RM Cappuzzo F Nishio M Mok TSK Reck M . Association of immune-related adverse events with efficacy of atezolizumab in patients with non-small cell lung cancer: pooled analyses of the phase 3 IMpower130, IMpower132, and IMpower150 randomized clinical trials. JAMA Oncol. (2023) 9:527–35. doi: 10.1001/jamaoncol.2022.7711, PMID: 36795388 PMC9936386

[6] Apalla Z Nikolaou V Fattore D Fabbrocini G Freites-Martinez A Sollena P . European recommendations for management of immune checkpoint inhibitors-derived dermatologic adverse events. The EADV task force ‘Dermatology for cancer patients’ position statement. J Eur Acad Dermatol Venereol: JEADV. (2022) 36:332–50. doi: 10.1111/jdv.17855, PMID: 34910332

[7] Peeraphatdit TB Wang J Odenwald MA Hu S Hart J Charlton MR . Hepatotoxicity from immune checkpoint inhibitors: A systematic review and management recommendation. Hepatol (Baltimore Md). (2020) 72:315–29. doi: 10.1002/hep.31227, PMID: 32167613

[8] Gupta S Cortazar FB Riella LV Leaf DE . Immune checkpoint inhibitor nephrotoxicity: update 2020. Kidney360. (2020) 1:130–40. doi: 10.34067/KID.0000852019, PMID: 35372904 PMC8809100

[9] Seethapathy H Zhao S Chute DF Zubiri L Oppong Y Strohbehn I . Causes, and risk factors of acute kidney injury in patients receiving immune checkpoint inhibitors. Clin J Am Soc Nephrol: CJASN. (2019) 14:1692–700. doi: 10.2215/CJN.00990119, PMID: 31672794 PMC6895474

[10] Manohar S Kompotiatis P Thongprayoon C Cheungpasitporn W Herrmann J Herrmann SM . Programmed cell death protein 1 inhibitor treatment is associated with acute kidney injury and hypocalcemia: meta-analysis. Nephrol Dialysis Transplant. (2019) 34:108–17. doi: 10.1093/ndt/gfy105, PMID: 29762725

[11] Weber JS Hodi FS Wolchok JD Topalian SL SChadendorf D Larkin J . Safety profile of nivolumab monotherapy: A pooled analysis of patients with advanced melanoma. J Clin Oncol. (2017) 35:785–92. doi: 10.1200/JCO.2015.66.1389, PMID: 28068177

[12] Cortazar FB Marrone KA Troxell ML Ralto KM Hoenig MP Brahmer JR . Clinicopathological features of acute kidney injury associated with immune checkpoint inhibitors. Kidney Int. (2016) 90:638–47. doi: 10.1016/j.kint.2016.04.008, PMID: 27282937 PMC4983464

[13] Naidoo J Murphy C Atkins MB Brahmer JR Champiat S Feltquate D . Society for Immunotherapy of Cancer (SITC) consensus definitions for immune checkpoint inhibitor-associated immune-related adverse events (irAEs) terminology. J Immunother Cancer. (2023) 11:e006398. doi: 10.1136/jitc-2022-006398, PMID: 37001909 PMC10069596

[14] Hasan Ali O Berner F Bomze D Fässler M Diem S Cozzio A . Human leukocyte antigen variation is associated with adverse events of checkpoint inhibitors. Eur J Cancer (Oxford England: 1990). (2019) 107:8–14. doi: 10.1016/j.ejca.2018.11.009, PMID: 30529903

[15] Iwama S De Remigis A Callahan MK Slovin SF Wolchok JD Caturegli P . Pituitary expression of CTLA-4 mediates hypophysitis secondary to administration of CTLA-4 blocking antibody. Sci Trans Med. (2014) 6:230ra45. doi: 10.1126/scitranslmed.3008002, PMID: 24695685

[16] Zhou P Gao Y Kong Z Wang J Si S Han W . Immune checkpoint inhibitors and acute kidney injury. Front Immunol. (2024) 15:1353339. doi: 10.3389/fimmu.2024.1353339, PMID: 38464524 PMC10920224

[17] Lee CL Riya IJ Piya IJ Muniz TP Butler MO Saibil SD . Immune checkpoint inhibitor-induced pancreatic injury (ICI-PI) in adult cancer patients: A systematic review and meta-analysis. Cancers (Basel). (2025) 17:1080. doi: 10.3390/cancers17071080, PMID: 40227596 PMC11987741

[18] Isik B Alexander MP Manohar S Vaughan L KottsChade L Markovic S . Biomarkers, clinical features, and rechallenge for immune checkpoint inhibitor renal immune-related adverse events. Kidney Int Rep. (2021) 6:1022–31. doi: 10.1016/j.ekir.2021.01.013, PMID: 33912752 PMC8071627

[19] De Martin E Michot JM Rosmorduc O Guettier C Samuel D . Liver toxicity as a limiting factor to the increasing use of immune checkpoint inhibitors. JHEP Rep. (2020) 2:100170. doi: 10.1016/j.jhepr.2020.100170, PMID: 33205034 PMC7648167

[20] Hountondji L Ferreira De Matos C Lebossé F Quantin X Lesage C Palassin P . Clinical pattern of checkpoint inhibitor-induced liver injury in a multicentre cohort. JHEP Rep. (2023) 5:100719. doi: 10.1016/j.jhepr.2023.100719, PMID: 37138674 PMC10149360

[21] EASL Clinical Practice Guidelines: Drug-induced liver injury. J Hepatol. (2019) 70:1222–61. doi: 10.1016/j.jhep.2019.02.014, PMID: 30926241

[22] De Martin E Michot JM Papouin B Champiat S Mateus C Lambotte O . Characterization of liver injury induced by cancer immunotherapy using immune checkpoint inhibitors. J Hepatol. (2018) 68:1181–90. doi: 10.1016/j.jhep.2018.01.033, PMID: 29427729

[23] Gauci ML Baroudjian B Zeboulon C Pages C Poté N Roux O . Immune-related hepatitis with immunotherapy: Are corticosteroids always needed? J Hepatol. (2018) 69:548–50. doi: 10.1016/j.jhep.2018.03.034, PMID: 29747956

[24] Onoyama T Takeda Y Yamashita T Hamamoto W Sakamoto Y Koda H . Programmed cell death-1 inhibitor-related sclerosing cholangitis: A systematic review. World J Gastroenterol. (2020) 26:353–65. doi: 10.3748/wjg.v26.i3.353, PMID: 31988594 PMC6969883

[25] Hwang SY Hsieh P Zhang W . Steroid-refractory immune checkpoint inhibitor (ICI) hepatitis and ICI rechallenge: A systematic review and meta-analysis. Hepatol Commun. (2024) 8:e0525. doi: 10.1097/HC9.0000000000000525, PMID: 39298568 PMC11412713

[26] Reeves HM Winters JL . The mechanisms of action of plasma exchange. Br J Haematol. (2014) 164:342–51. doi: 10.1111/bjh.12629, PMID: 24172059

[27] Kubo T Sugawara T Shinkawa T Kurisu T Kouzen N Tanaka T . Fatal fulminant hepatitis induced by combined ipilimumab and nivolumab therapy despite favorable histologic response and confirmed by autopsy in a patient with clear cell renal cell carcinoma. Immunol Med. (2021) 44:136–41. doi: 10.1080/25785826.2020.1788229, PMID: 32634346

[28] Riveiro-Barciela M Muñoz-Couselo E Fernandez-Sojo J Diaz-Mejia N Parra-López R Buti M . Acute liver failure due to immune-mediated hepatitis successfully managed with plasma exchange: New settings call for new treatment strategies? J Hepatol. (2019) 70:564–6. doi: 10.1016/j.jhep.2018.10.020, PMID: 30503040

[29] Chen G Wu M Wu B Liu F Liu J Liu L . Effects of dual plasma molecular adsorption system on liver function, electrolytes, inflammation, and immunity in patients with chronic severe hepatitis. J Clin Lab Anal. (2019) 33:e22926. doi: 10.1002/jcla.22926, PMID: 31206768 PMC6757123

[30] Yan GS Li LL Jiang SL Meng S Wu CC . Clinical study of different adsorbents with dual plasma molecular adsorption system in the treatment of hepatic failure. Zhonghua Gan Zang Bing Za Zhi = Zhonghua Ganzangbing Zazhi = Chin J Hepatol. (2019) 27:51–5. doi: 10.3760/cma.j.issn.1007-3418.2019.01.011, PMID: 30685924 PMC12770032

[31] Verzoni E Cartenì G Cortesi E Giannarelli D De Giglio A Sabbatini R . Real-world efficacy and safety of nivolumab in previously-treated metastatic renal cell carcinoma, and association between immune-related adverse events and survival: the Italian expanded access program. J Immunother Cancer. (2019) 7:99. doi: 10.1186/s40425-019-0579-z, PMID: 30944023 PMC6448290

[32] Zhong L Wu Q Chen F Liu J Xie X . Immune-related adverse events: promising predictors for efficacy of immune checkpoint inhibitors. Cancer Immunol Immunother: CII. (2021) 70:2559–76. doi: 10.1007/s00262-020-02803-5, PMID: 33576872 PMC10991616

[33] Liu Q Liu M Zou Z Lin J Zhang N Zhao L . Tofacitinib for the treatment of immune-related adverse events in cancer immunotherapy: a multi-center observational study. J Trans Med. (2024) 22:803. doi: 10.1186/s12967-024-05617-6, PMID: 39210332 PMC11360683

[34] Wang M Reynolds KL Montazeri K Schaefer EA Sullivan RJ Dougan M . Tofacitinib is effective in treating refractory immune checkpoint inhibitor hepatitis. Clin Gastroenterol Hepatol. (2024) 22:1539–1541.e2. doi: 10.1016/j.cgh.2023.12.011, PMID: 38142835 PMC12817493

