# Supplementary figures and images for: Case report of immune checkpoint inhibitor induced cholestatic hepatitis, acute renal injury and asymptomatic pancreatic enzyme elevation simultaneously
Source: Front Immunol. 2025 Nov 19;16:1679328. doi: 10.3389/fimmu.2025.1679328 (PMC12672487; doi:10.3389/fimmu.2025.1679328)

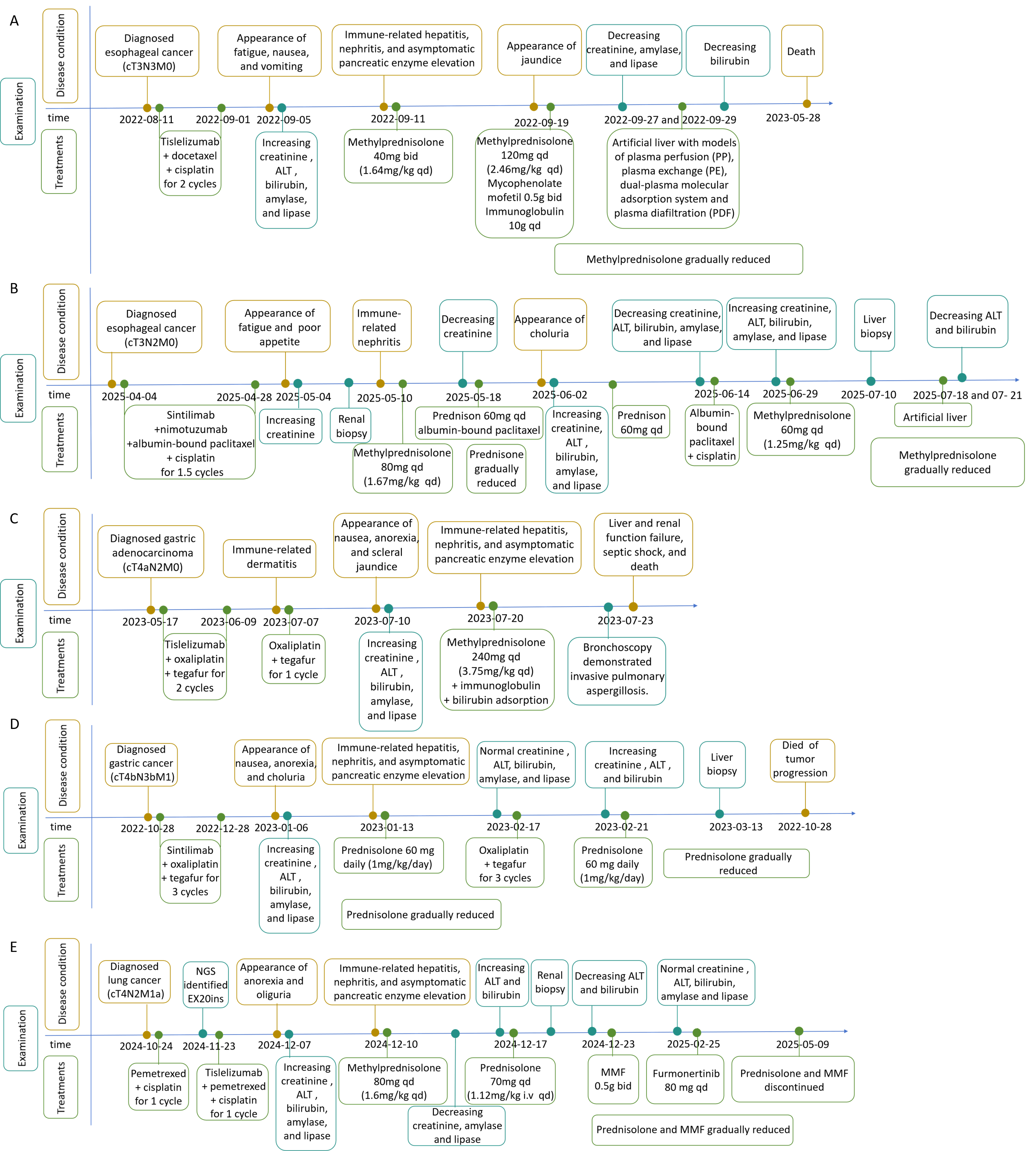

Supplement: Supplementary Figure 1 — Timeline for disease onset, treatment interventions, and outcomes of (A) Case 1, (B) Case 2, (C) Case 3, (D) Case 4 and (E) Case 5. [file Image1.tif]
